# Association between bodyweight perception, nutritional status, and weight control practices: A cross-sectional analysis from the Chilean Health Survey 2016–2017

**DOI:** 10.3389/fpsyg.2022.984106

**Published:** 2022-09-27

**Authors:** Gabriela Nazar, Carlos-María Alcover, Fabián Lanuza, Ana María Labraña, Karina Ramírez-Alarcón, Claudia Troncoso-Pantoja, Ana María Leiva, Carlos Celis-Morales, Fanny Petermann-Rocha

**Affiliations:** ^1^Departamento de Psicología y Centro de Vida Saludable, Universidad de Concepción, Concepción, Chile; ^2^Departamento de Psicología, Facultad de Ciencias de la Salud, Universidad Rey Juan Carlos, Madrid, Spain; ^3^Biomarkers and Nutrimetabolomics Laboratory, Department of Nutrition, Food Sciences and Gastronomy, Food Technology Reference Net (XIA), Faculty of Pharmacy and Food Sciences, Nutrition and Food Safety Research Institute (INSA), University of Barcelona, Barcelona, Spain; ^4^Facultad de Medicina, Centro de Epidemiología Cardiovascular y Nutricional (EPICYN), Facultad de Medicina, Universidad de La Frontera, Temuco, Chile; ^5^Departamento de Nutrición y Dietética, Facultad de Farmacia, Universidad de Concepción, Concepción, Chile; ^6^Departamento de Salud Pública, Facultad de Medicina, Centro de Investigación en Educación y Desarrollo (CIEDE-UCSC), Universidad Católica de la Santísima Concepción, Concepción, Chile; ^7^Facultad de Medicina, Instituto de Anatomía, Histología y Patología, Universidad Austral, Valdivia, Chile; ^8^BHF Glasgow Cardiovascular Research Centre, School of Cardiovascular & Metabolic Health, University of Glasgow, Glasgow, United Kingdom; ^9^Laboratorio de Rendimiento Humano, Grupo de Estudio en Educación, Actividad Física y Salud (GEEAFyS), Universidad Católica del Maule, Talca, Chile; ^10^Facultad de Medicina, Centro de Investigación Biomédica, Universidad Diego Portales, Santiago, Chile

**Keywords:** body weight, body image, obesity, weight loss, weight perception

## Abstract

This research aimed (1) to examine the agreement between body mass index (BMI)-based nutritional status and perceived nutritional status overall and by socio-demographic factors and (2) to state the association between the accuracy of weight perception and weight control practices in the Chilean adult population. A population-based cross-sectional study was carried out with 5,192 Chilean adult participants from the Chilean National Health Survey 2016–2017. Agreement between BMI-based weight status and body weight perception for the total sample and across subgroups was determined using the weighted kappa coefficient. The agreement between BMI-based and perceived nutritional status of the total sample was fair (kappa = 0.38). A higher rate of weight perception accuracy was identified in women, younger respondents, and participants with higher education, a higher income, and from urban areas than their counterparts. Respondents with overweight or obesity tended to underestimate their nutritional status. Actions to lose weight were higher in those who had the right perception of their overweight/obesity condition and those who overestimated their body weight, regardless of their nutritional status. In all groups, weight loss behaviors were more related to the perceived than the BMI-based nutritional status. The consequences of accurate perception of the nutritional status are discussed including its effects on body weight and mental health.

## Background

Obesity is considered one of the main causes of chronic diseases and one of the leading causes of global health burden (World Health Organization, 2022). Despite the efforts, obesity rates continue growing, particularly in developing countries, which imposes a critical challenge for governments, health systems, stakeholders, and society as a whole (OECD Obesity Update, 2017; World Health Organization, 2022).

Some psychological factors, such as beliefs and internal evaluative processes about body image, have important consequences on health (McCabe et al., 2006; Gillen, 2015). Among these processes, body weight perception—or how a person regards their body weight—has been widely studied due to its consequences on eating behavior, obesity, health outcomes, and mental health (McCabe et al., 2006; Gillen, 2015; Pool et al., 2019; Darimont et al., 2020). Body weight perception is usually determined compared with individuals’ nutritional status based on body mass index (BMI) (Howard et al., 2008). In other words, people can classify themselves as either underweight, normal weight, overweight, or obese, having the right perception, underestimating, or overestimating their nutritional status (Park, 2011).

Evidence shows discrepancies between self-perceived and BMI-based nutritional status, with differences according to persons’ socio-demographic characteristics, particularly depending on their current body weight status. For instance, studies informed that around 30–50% of the samples studied underestimated their weight (Dorsey et al., 2009; Duncan et al., 2011; Hassan et al., 2018). These figures increase in overweight or obese people (Dorsey et al., 2009), men (Alwan et al., 2010), people from low-income levels (Hassan et al., 2018), and those with low educational attainment (Squiers et al., 2014; Hassan et al., 2018).

The perceived nutritional status is relevant since it can act as a facilitator or constraint to initiating and maintaining weight control practices (Duncan et al., 2011; Yaemsiri et al., 2011; Hassan et al., 2018; Haynes et al., 2018). The risks of undetected obesity have been reported (Robinson et al., 2017), and the traditional assumption is that individuals’ failure to accurately identify their weight condition influences weight management strategies. Accordingly, people who recognize weight problems are prone to implement actions to lose weight (Pool et al., 2019).

Weight perception has a sociocultural component since there are social norms, values, beliefs, and expectations that inform about body size ideals and what is considered a normal or healthy body weight (Wardle et al., 2001; Mellor et al., 2008; Christoph et al., 2018; Robinovich et al., 2018). Therefore, it is necessary to analyze this phenomenon and its consequences in a contextualized and country-specific manner. Chile, where this research took place, has been facing an increase in obesity rates due to the fast economic development and nutritional transition experienced by the population during the past four decades (Petermann-Rocha et al., 2020). Therefore, quantifying the levels of agreement between perceived and measured nutritional status in the population could inform future public health policies aiming to tackle the current obesity prevalence in this country (Ministerio de Salud Chile, 2022). Then, this study aimed (1) to examine the accuracy between BMI-based nutritional status and perceived nutritional status overall and by socio-demographic factors and (2) to state the association between the accuracy of weight perception and weight control behavior in the Chilean adult population.

## Materials and methods

This is a cross-sectional study that used data from the Chilean National Health Survey 2016–2017 (CNHS, 2016–2017) (Ministerio de Salud, 2017). The CNHS was a survey carried out in a probabilistic, stratified, and multistage sample of 6,233 people ≥ 15 years, with national and geographical representativeness. This study employed data from a subsample of 5,192 people ≥ 18 years, 63.9% women, mean age of 44.9 years, and who had available data in the variables of interest.

Protocols of the CNHS 2016–2017 received approval from the Ethics Committee of the Medicine School at the Pontificia Universidad Católica de Chile (Ministerio de Salud, 2017).

### Variables and instruments

Nutritional status was defined according to the BMI [weight (kg)/height (m)^2^]. Bodyweight (kg) and height (m) were measured by trained nurses using standardized protocols, and nutritional status was derived from BMI categorized as underweight (BMI < 18.5 kg/m^2^), normal weight (18.5 ≥ BMI < 25 kg/m^2^), overweight (25 ≥ BMI < 30 kg/m^2^), and obese (BMI ≥ 30 kg/m^2^) for the population aged 18 to < 60 years of age (WHO Consultation on Obesity, 1997). For older people (≥ 60), the Pan American Health Organization (PAHO) criteria were applied (underweight: < 23.0 kg/m^2^; normal: 23.0–27.9 kg/m^2^; overweight: 28.0–31.9 kg/m^2^; obese: ≥ 32.0 kg/m^2^) (PAHO, 2003).

Bodyweight perception was assessed through the following question*: “You consider yourself as: (*people were shown a card with the following statements). (*1) Underweight; (2) Normal weight; (3) Overweight; (4) Obese*.” Then, three categories were created using the nutritional status and body weight perception categories as follows: (i) people with an accurate weight perception or those whose body weight perception was the same as their BMI-based nutritional status category; (ii) people that underestimated their weight or those who perceived their body weight as lower than their BMI-based category; and (iii) people who overestimated their weight or perceived their body weight as more elevated than their BMI-based category.

Weight control management was evaluated with the following two questions: (a) *“have you been dieting on your own to lose weight in the last 2 weeks?”* and (b) *“have you been exercising regularly to lose weight in the last 2 weeks*?” Answer options were: (a) *Yes* or (b) *No.*

*Socio-demographic data* included sex (men or women), educational level (high, medium, and low), residential area (rural or urban), and socioeconomic status [SES] (low, medium, and high). These data were obtained from validated self-reported questionnaires from the CNHS 2016–2017 (Ministerio de Salud, 2017).

### Statistics analyses

Descriptive statistics were carried out using percentages and mean with their respective 95% confidence intervals (95% CI). The degree of agreement between BMI-based weight status and body weight perception for the total sample and across subgroups by weight status and socio-demographic factors was determined using the weighted Kappa coefficient. The strength of agreement depends on *K* value, according to the following classification: < 0.20 = poor; 0.21–0.40 = fair; 0.41–0.60 = moderate, 0.61–0.80 = good, and 0.81–1.00 = very good (McHugh, 2012). To explore the association between the accuracy of weight perception and weight control practices, Poisson regression analyses were performed because they provide prevalence ratio (PR) estimates that are easy to interpret (Grant, 2014). Results were reported as PR with their 95% CIs. These analyses were adjusted for age, sex, zone of residency, education, and BMI-based nutritional status.

All analyses were estimated using expanded samples (svy) from the CNHS 2016–2017 (Ministerio de Salud, 2017) using the StataMP version 17 software. Significance differences were set up at *p* < 0.05.

## Results

Characteristics of the total sample according to their BMI-based nutritional status are shown in Table 1. According to BMI-based nutritional status, 26.7% of the participant were classified as normal weight, while nearly 70% presented as overweight or obese. Overall, underweight individuals were older, while those with a normal BMI were younger.

**TABLE 1:** Sample characteristics by BMI-based nutritional status.

BMI-based nutritional status	Total sample	Underweight	Normal	Overweight	Obesity
*n* (%)	5,192 (100)	200 (3.85)	1,385 (26.7)	1,861 (35.8)	1,746 (33.6)
*n* expanded	13,356,998	368,431	3,361,752	5,256,477	4,370,338
Age (mean) [95% CI]	44.9 (44.2–45.7)	61.2 (55.6–67.0)	43.6 (41.9–45.5)	44.6 (43.4–45.9)	44.8 (43.7–46.0)
Weight (mean)	76.2 (75.5–77.0)	50.7 (48.9–52.5)	62.97 (62.1–63.8)	74.85 (74.1–75.6)	90.22 (89.1–91.4)
Height (cm)	1.62 (1.62–1.63)	158.4 (156.4–160.4)	163.1 (162.1–164.0)	163.6 (162.9–164.4)	161.3 (160.4–162.2)
BMI (mean)	28.82 (28.5–29.0)	20.2 (19.6–20.7)	23.6 (23.4–23.8)	27.9 (27.8–28.0)	34.7 (34.3–35.0)

BMI, body mass index (kg/m^2)^). Underweight: aged 18–60 years: BMI < 18.5. Age: ≥ 60 years: BMI < 23. **Normal weight:** aged 18–60 years: 18.5 ≥ BMI < 25, ≥ 60 years old: 23 ≥ BMI < 28. Overweight: aged 18–60 years: 25 ≥ BMI < 30, ≥ 60 years old: ≥ 28 BMI < 32. Obese: aged 18–60 years: BMI ≥ 30, ≥ 60 years: BMI ≥ 32 (Ministerio de Salud Chile, 2010).

The proportion of participants according to their BMI-based nutritional status and body weight perception is shown on the left side of Table 2. From the total sample, 5.3% perceived themselves as underweight, 41.0% as normal weight, 48.0% as overweight, and 5.7% as self-perceived as obese. The same data are shown according to socio-demographic characteristics.

**TABLE 2:** BMI-based nutritional status, perceived nutritional status, accuracy of weight perception, and level of agreement between BMI-based weight status and weight perception, in the total sample and by socio-demographic characteristics.

	Self-perception of nutritional status (%)				Accuracy of weight perception and degree of agreement between BMI-based weight status an < weight perception (%)			
		
BMI-based weight status	Under- weight	Normal weight	Over- weight	Obese	Right (%)	Underestimate	Overestimate (%)	Kappa coefficient
**Total Sample** (n = 5,192)	5.28	41.0	48.0	5.7	49.81	45.20	4.99	0.38 (**)
Underweight (n = 200)	55.8 (44.2–66.8)	43.2 (32.4–54.8)	0.9 (0.2–3.2)	0.0 (0.0–0.0)	55.8 (44.3)		44.2 (33.1–55.7)	
Normal weight (n = l,385)	12.9 (9.7–17.0)	73.3 (68.7–77.5)	13.5 (10.5–17.1)	0.2 (0.0–1.2)	73.3 (68.6–77.5)	12.9 (9.7–17.0)	13.7 (10.7–17.4)	
Overweight (n = l,861)	1.3 (0.8–2.2)	36.6 (32.9–40.5)	61.2 (57.3–65.0)	0.8 (0.0–1.5)	61.2 (57.3–65.0)	37.9 (34.2–41.8)	0.8 (0.4–1.5)	
Obese (n = l,746)	0.0 (0.0–0.1)	8.0 (6.0–10.6)	74.5 (70.6–78.0)	17.5 (14.5–20.8)	17.5 (14.5–20.8)	82.5 (79.1–85.5)	**–**	
**Women** **(n = 3,318)**	4.7	36.8	51.4	7.2	51.8	42.5	5.7	0.39 (**)
Underweight	61.0 (45.4–74.1)	39.0 (25.7–54.2)	0.3 (0.0–1.2)	0.0 **–**	61.0 (45.4–74.1)	**–**	39.3 (25.9–54.5)	
Normal weight	10.5 (6.9–15.4)	72.1 (65.8–77.6)	17.5 (13.0–23.0)	0.0	72.1 (65.9–77.5)	10.5 (6.9–15.4)	17.5 13.0–23.0)	
Overweight	1.4 (0.6–2.8)	29.3 (24.9–34.2)	67.9 (63.0–72.5)	1.3 (0.6–2.8)	67.9 (63.0–72.5)	30.7 (26.2–35.6)	1.3 (0.6–2.7)	
Obese	0.0 (0.0–0.2)	6.5 (4.2–9.8)	71.3 (66.4–75.9)	22.1 (18.0–26.7)	22.1	77.9	**–**	
**Men** **(n = 1874)**	6.4	48.6	41.9	3.5	47.8	48.0	4.2	0.35 (**)
Underweight	50.2 (33.6–66.8)	48.2 (31.8–65.0)	1.6 (0.3–0.7)	0.0 **–**	50.2 (33.6–66.8)		49.78 (33.2–66.4)	
Normal weight	15.2 (10.3–21.9)	74.4 (67.3–80.4)	9.9 (6.3–15.1)	0.4 (0.0–2.2)	74.4 (67.3–80.4)	15.2 (10.3–21.9)	10.3 (6.7–15.6)	
Overweight	1.3 (0.0–2.6)	43.2 (37.5–49.1)	55.1 (49.2–60.9)	0.3 (0.0–1.2)	55.1 (49.2–60.9) 11.1	44.5 (38.7–50.4) 88.8	0.3 (0.1–1.2)	
Obese	0.0	10.0 (6.7–14.8)	78.8 (72.7–83.8)	11.1 (7.5–16.2)	(7.5–16.2)	(0.84–92.5)		
**Age group** ** < 60** **(n = 3,426)**	4.1	37.0	51.7	7.3	49.0	47.5	3.5	0.35 (**)
Underweight	83.0 (62.8–93.4)	16.6 (6.3–36.8)	0.0 (0.0–3.1)	0.0 0.0–1.6	83.0 (62.8–93.4)	0	17.0 (6.6–37.1)	
Normal weight	14.6 (10.6–19.8)	72.7 (66.8–77.8)	12.4 (9.0–16.9)	0.0 0.0–1.9	72.7 (66.8–77.8)	14.6 (10.6–19.8)	12.8 (9.3–17.2)	
Overweight	1.0 (0.4–1.9)	36.8 (32.5–41.4)	61.3 (56.7–65.6)	0.1 0.4–1.9	61.3 (56.7–65.6)	37.8 (33.5–42.4)	0.9 (0.4–1.9)	
Obese	0.0 (0.0–0.1)	7.9 (5.6–11.0)	73.1 (68.7–77.1)	18.9 15.5–22.8	18.9 (15.5–22.9)	81.1 (77.1–84.5)	0 **–**	
** > 60** **(n = l,766)**	7.6	48.9	40.8	2.7	52.6	37.1	10.3	0.40 (**)
Underweight	47.0 (35.1–59.2)	52.0 (39.8–63.9)	1.1 (0.0–4.3)	0.0 **–**	47.0 (35.1–59.2)	0 **–**	53.0 (40.1–15.8)	
Normal weight	8.9 (4.8–15.8)	75.0 (67.1–81.5)	16.2 (11.0–23.2)	0.0 **∼**	75.0 (67.0–81.6)	8.9 (4.8–15.8)	16.2 (10.9–23.1)	
Overweight	2.9 (1.3–5.8)	35.6 (29.3–42.5)	61.2 (54.2–67.8)	0.3 (0.1–0.8)	61.2 (54.2–67.8)	38.5 (31.9–45.5)	0.3 0.1–0.8)	
Obese	0.0 **–**	8.5 (5.8–12.3)	82.0 (76.5–86.5)	9.4 (6.2–13.9)	9.4 (6.3–13.9)	90.6 (86.1–93.7)		
**Education** **level** **Low** **(n = l,287)**	7.7	44.6	44.1	3.6	39.8	53.6	6.6	0.34 (**)
Underweight	47.3 (31.4–63.8)	52.3 (35.9–68.3)	0.0 (0.0–1.5)	**–**	47.3 (31.4–63.8)	**–**	52.7 (36.2–68.6)	
Normal weight	12.9 (6.4–24.3)	75.6 (64.4–94.2)	11.4 (6.3–19.5)	**–**	75.6 (64.4–84.1)	13.0 (6.4–24.3)	11.4 (6.3–19.8)	
Overweight	5.0 (2.6–9.4)	46.5 (38.2–54.9)	48.1 (39.8–56.5)	0.3 (0.1–1.2)	48.1 (39.8–56.5)	51.5 (43.1–59.8)	0.4 (0.0–1.2)	
Obese	0.0 (0.0–0.2)	15.6 (9.7–24.2)	74.4 (66.1–81.3)	9.8 (6.5–14.5)	9.8 (6.5–14.5)	90.2 (85.5–93.5)	**–**	
**Medium** **(n = 2,716)**	4.8	39.9	48.7	6.5	48.1	47.1	4.8	0.36 (**)
Underweight	65.3 (48.2–79.1)	33.1 (19.6–50.2)	1.6 (0.3–7.0)	0.0	65.3 (48.2–79.1)	**–**	34.7 (20.9–51.8)	
Normal weight	13.7 (9.5–19.4)	70.3 (63.3–76.4)	16.0 (11.1–22.3)	0.0 **–**	70.3 (63.3–76.4)	13.7 (9.5–19.4)	16.0 (11.1–22.3)	
Overweight	1.0 (0.0–2.4)	39.4 (34.4–44.6)	58.6 (53.4–63.6)	0.9 (0.3–2.2)	58.6 (53.4–63.6)	40.5 (35.5–45.7)	0.9 (0.3–2.2)	
Obese	0.0 (0.0–0.3)	5.7 (4.0–7.9)	74.5 (69.4–78.9)	19.8 (15.6–24.7)	19.8 (15.7–24.8)	80.2 (75.2–84–3)		
**High** **(n = l,189)**	3.6	39.8	50.4	6.2	58.7	36.9	4.4	0.48 (***)
Underweight	46.4 (20.4–74.5)	53.6 (25.4–79.6)	0.0 **–**	0.0 **–**	46.4 (20.4–74.5)	**–**	53.6 (25.4–79.6)	
Normal weight	11.9 (6.7–20.2)	76.2 (68.0–82.9)	11.2 (7.4–16.6)	0.7 (0.1–3.2)	76.2 (68.0–82.9)	11.9 (7.9–17.3)	11.9 (7.9–17.3)	
Overweight	0.0 (0.0–1.5)	27.7 (21.0–35.5)	71.2 (63.4–78.0)	0.8 (0.3–2.1)	71.2 (63.4–78.0)	27.9 (21.2–35.8)	0.8 (0.2–2.1)	
Obese	**–**	6.7 (2.9–15.1)	74.7 (65.5–82.1)	18.5 (12.4–26.7)	18.5 (12.4–22.7)	81.5 (73.3–87.59		
**Residential** **Area**								
**Urban** **(n = 4,343)**	5.4	40.8	47.9	5.9	51.1	44.1	4.8	0.39 (**)
Underweight	61.7 (49.0–72.9)	37.3 (26.2–50.0)	0.9 (0.2–4.0)	0.0 **–**	61.7 (49.0–72.9)	**–**	38.3 (27.1–50.9)	
Normal weight	13.0 (9.6–17.5)	73.3 (68.2–77.8)	13.4 (10.1–17.4)	0.2 (0.0–1.3)	73.3 (68.2–77.8)	13.1 (9.6–17.5)	13.6 (10.4–17.6)	
Overweight	1.3 (0.8–2.3)	34.9 (30.9–39.0)	62.8 (58.7–66.8)	0.8 (0.5–1.7)	62.8 (58.7–66.8)	36.3 (32.2–40.4)	0.9 (0.4–1.7)	
Obese	0.0 (0.0–0.1)	7.5 (5.4–10.6)	74.8 (70.5–78.7)	17.5 (14.3–21.4)	17.5 (14.3–21.4)	82.5 (78.6–85.7)		
**Rural** **(n = 834)**	4.6	42.2	48.3	4.9	39.3	54.3	6.3	0.32 (**)
Underweight	29.1 (12.7–53.6)	70.4 (45.9–86.9)	0.5 (0.0–4.1)	0.0 **–**	29.1 (12.7–53.6)	**–**	70.9 (46.3–87.3)	
Normal weight	11.5 (5.9–21.1)	73.9 (63.7–82.1)	14.6 (9.1–22.4)	0.0 **–**	73.9 (63.7–82.1)	11.5 (5.9–21.1)	14.6 (9.1–22.4)	
Overweight	1.4 (0.3–5.6)	52.9 (43.9–61.8)	45.6 (36.9–54.7)	0.0 **–**	45.6 (36.9–54.7)	54.4 (45.3–63.1)	**–**	
Obese	0	10.7 (6.6–16.9)	72.4 (64.7–79.0)	16.9 (11.7–23.7)	16.9 (11.7–23.7)	83.1 (76.2–88.3)	**–**	
**Socioecono** **mic level** **Low** **(n = l,601**	6.8	41.5	46.3	5.4	44.7	50.3	5.0	0.36 (**)
Underweight	51.6 (35.4–67.6)	46.5 (30.9–62.8)	1.8 (0.3–0.9)	0.0	51.6 (35.4–67.6)		48.4 (32.2–64.6)	
Normal weight	15.4 (10.3–22–4)	74.7 (67.1–81.0)	9.9 (6.4–15.0)	0.0 **–**	74.7 (67.1–81.0)	15.4 (10.3–22.4)	9.9 (6.4–15.0)	
Overweight	3.3 (1.7–6.5)	43.2 (36.0–50.5)	52.7 (45.3–60.0)	0.8 (0.0–1.8)	52.7 (45.3–60.0)	46.5 (39.2–53.9)	0.8 (0.3–1.8)	
Obese	0.1 (0.0–0.7)	11.3 (6.7–17.6)	71.5 (63.9–78.0)	17.4 (12.3–24.0)	17.4 (12.3–24.0)	82.6 (76.0–87.7)		
**Medium** **(n = l,285)**	5.7	40.2	47.4	6.8	45.9	48.7		5.4 0.37 (**)
Underweight	59.1 (37.6–77.6)	40.4 (22.0–62.0)	0.4 (0.0–1.8)	0.0 **–**	59.1 (37.6–77.6)	**–**	40.9 (22.4–62.4)	
Normal weight	16.7 (9.8–27.0)	68.9 (58.6–77.7)	14.4 (8.6–23.0)	0.0 **–**	68.9 (58.6–77.7)	16.7 (9.8–27.0)	14.4 (8.6–23.0)	
Overweight	1.1 (0.3–4.0)	42.5 (34.5–50.7)	55.6 (47.3–63.5)	0.8 (0.2–2.3)	55.6 (47.3–63.5)	43.7 (35.7–51.9)	0.8 (0.2–2.3)	
Obese	0.0 (0.0–0.1)	8.9 (5.4–14.2)	71.0 (63.3–77.6)	20.1 (14.4–27.4)	20.1 (14.4–27.4)	79.9 (72.6–85–6)	**–**	
**High (n = 1387)**	3.2	39.4	51.5	5.9	54.0	41.0	5.0	0.39* (**)
Underweight	43.2 (21.8–67.5)	56.2 (32.0–77.8)	0.6 (0.0–4.1)	0.0 **–**	43.2 (21.8–67.5)	**–**	56.8 (32.5–78.1)	
Normal weight	9.8 (5.2–6.7)	73.1 (64.4–80.3)	16.4 (11.0–23.7)	0.6 (0.0–3.4)	73.1 (64.4–80.3)	9.7 (5.2–17.5)	17.1 (11.6–24.4)	
Overweight	0.3 (0.0–1.0)	29.8 (24.1–36.3)	69.4 (62.9–75.2)	0.5 (0.1–1.8)	69.4 (62.9–75.2)	30.1 (24.3–36.5)	0.5 (0.1–1.8)	
Obese	0.0 (0.0–0.2)	6.3 (3.2–12.3)	77.9 (70.6–83.8)	15.7 (11.0–22.1)	15.7 (11.0–22.1)	84.3 (77.9–89.0)		

The right side of Table 2 presents the percentage of participants according to the accuracy of their weight perception. From the total sample, 49.8% of participants perceived their nutritional status correctly, while 45.2% underestimated and 5.0% overestimated it.

The level of agreement between BMI-based and perceived body weight for the total sample was 0.38, i.e., classified as *fair* strength of agreement. In the analysis of subgroups, participants with higher agreement levels were those with the highest educational degree (kappa = 0.48). On the contrary, participants with the lowest educational degree, the lowest SES, and those from rural areas showed the lowest level of agreement. In terms of subcategories, women than men (kappa = 0.39 vs. kappa = 0.35) and participants older than 60 years than those < 60 (kappa = 0.40 vs. kappa = 0.35) showed higher agreement.

In the analysis of the accuracy between participants’ weight perception and their current weight, data indicated that participants classified as *normal weight* according to their BMI showed a higher percentage of accuracy than those classified as underweight, overweight, and obese. These levels of agreement were similar across age, sex, educational status, SES, and residential area (urban/rural) (Table 2). Since there are no categories of nutritional status that are above the perception of obesity (e.g., morbid obesity), participants with obesity cannot overestimate their nutritional status. Notwithstanding the foregoing, 82.5% of participants classified as obese underestimated their current body weight. This percentage increased to 88.8% in men, 90.6% in people aged ≥ 60, and 90.2% in the group with the lowest education level.

Regarding weight management strategies, results indicated that participants who followed a diet in the past 2 weeks and/or exercised to lose weight were more likely to be obese according to their BMI-based status and also those who overestimated their body weight regardless of their current nutritional status (Table 3).

**TABLE 3:** Weight management practices in the total sample according to their BMI-based nutritional status and accuracy of weight perception.

BMI-based nutritional status	Have followed a diet on their own to lose weight in the past 2 weeks (%, 95% CI)	Have exercised to lose weight during the last 2 weeks (%, 95% CI)
Underweight	2.3 (0.7–7.4)	0.0
Normal weight	8.3 (6.0–11.6)	12.0 (8.8–16.2)
Overweight	22.9 (19.5–26.7)	11.0 (8.8–13.8)
Obese	29.7 (19.5–26.7)	13.9 (11.0–17.3)
Accurate body weight perception	20.0 (17.2–23.2)	12.2 (10.0–15.0)
Underestimate body weight	21.3 (18.5–24.4)	11.1 (8.9–13.7)
Overestimate body weight	25.9 (17.6–36.4)	15.8 (9.1–26.1)

When comparing the weight management practices according to the BMI-based nutritional status and accuracy of weight perception (Table 4), data showed that the highest proportion of participants dieting and exercising to lose weight were those in the normal weight according to BMI who also overestimated their weight (34.8% followed a diet and 21.9% exercised to lose weight). The percentage of normal-weight participants who dieted was more than 7 times higher than those who overestimated their body weight compared with those with a right perception of their weight (34.8 vs. 4.9%).

**TABLE 4:** Frequency (%) of weight management practices based on BMI-based nutritional status and accuracy of the weight perception.

	Right perception		Underestimate their nutritional status		Overestimate their nutritional status	
BMI-based weight status	Have followed a diet to lose weight	Have exercised to lose weight	Have followed a diet to lose weight	Have exercised to lose weight	Have followed a diet to lose weight	Have exercised to lose weight
Underweight	3.2 (0.7-13.5)	0	–	–	1.3 (0.2-5.7)	–
Normal weight	4.9 (2.9-8.0)	12.3 (8.5-17.5)	0.4 (0.1-1.8)	0.2 (0.0-0.9)	34.8 (23.4-48.3)	21.9 (12.4-35.6)
Overweight	29.8 (24.9-35.2)	12.3 (9.3-16.0)	11.7 (8.2-16.3)	9.0 (0.6-13.3)	23.9 (7.1-56.5)	11.2 (2.5-37.8)
Obese	32.2 (23.8-42.0)	15.3 (9.2-24.4)	29.2 (25.1-33.6)	13.5 (10.5-17.4)	–	—

In the group classified as overweight, 11.7% of participants who underestimated their weight reported dieting, and this percentage increased to 29.8% for those with a right weight perception and to 23.9% for those who overestimated their weight. In the group with obesity, 32.21% of participants with right weight perception and 29.19% of those who underestimated their weight had followed a diet to lose weight. Those who exercised to lose weight were more likely normal-weight people who overestimated their weight.

**FIGURE 1:**
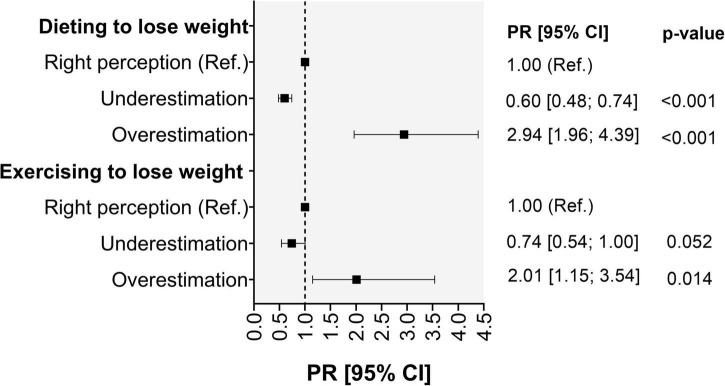
Association between the accuracy of weight perception and weight control practices. PR, Poisson Regression.

The associations between the accuracy of weight perception and weight control practices are shown in Figure 1. Compared with individuals with the right perception of their nutrition status, those who underestimated it had a lower likelihood of following a diet [PR: 0.60 (95% CI: 0.48–0.74)] or exercising to lose weight, even if the latter was non-statistically significant. On the contrary, compared with those with the right perception of their nutritional status, participants who overestimated their nutritional status showed a higher likelihood of following a diet [PR = 2.94 (95% CI: 1.96–4.39)] or exercising [RP = 2.01(95% CI: 1.15–3.54)] (Figure 1).

## Discussion

The main results indicated that only half of the total sample perceived their nutritional status correctly, and among those who misperceived their condition, the great majority tended to underestimate their nutritional status. Even if the agreement level was fair, accuracy was higher in normal-weight people compared with the groups classified as overweight or obese. Additionally, accuracy increased in women, urban, and those with higher educational levels and higher SES than their counterparts.

A recent systematic review shows wide heterogeneity in the rate of accurate weight self-perception, which varied between 16 and 83%, being less common in American and African populations, and representative samples of the US and Mexican people compared with Europeans (Freigang et al., 2020). This misperception might suggest the influence of cultural factors, social norms, or comparisons within the close environment (Wardle et al., 2001).

There is a consensus that normal-weight people are more likely to correctly categorize their nutritional status than overweight or obese participants (Christoph et al., 2018; Freigang et al., 2020; Dey et al., 2019). Moreover, underestimating the BMI categories tends to be higher in people with excessive weight (Robinson and Oldham, 2016; Dey et al., 2019; Freigang et al., 2020). In research with almost 1,800 participants from eastern Caribbean countries, 54% of overweight and 23% of obese participants underestimated their actual body weight compared with 30% in the whole sample (Hassan et al., 2018). Another research using data from a UK population-based study informed that almost one-third of women (31%) and 55% of men overweight classified their nutritional status as being “about right” (Robinson and Oldham, 2016).

To explain the underestimation of obesity, Robinson et al. (2017) proposed a visual normalization theory based on the notion that weight status is judged relative to visual body size norms, and those body size norms are determined by the body size of people who are usually exposed to them. These images ended up being considered the *norm* to assess one’s and others’ body sizes. In the case of the Chilean population, high rates of overweight and obesity have been reported (Celis-Morales et al., 2017). The last CNHS 2016–2017 informed that the percentage of the population classified as overweight, obese, and morbid obesity increased from 64.4 to 74.2% from the CNHS 2009–2010 (Ministerio de Salud, 2017). These data suggest that weight excess has become normal for Chilean people and so it is undetected.

Women tended to perceive their nutritional status more accurately than men, the same as reported in multiple studies, from different age groups and cultural contexts (Howard et al., 2008; Alwan et al., 2010; Park, 2011; Yaemsiri et al., 2011). Women usually show concerns about their body image associated with social demands that induce women, more than men, to be aware of their physical appearance and body weight (Holmqvist and Frisén, 2010; Frederick et al., 2022). This experience may prompt women to be more vigilant of their size and weight, resulting in more accuracy in their weight perception. Notwithstanding the above, data from a Danish study that examined changes in the prevalence of overweight and weight misperception among overweight people between 1995 and 2008 informed a reduction in the proportion of overweight men misperceiving their weight (Matthiessen et al., 2014), or even no differences between sexes (Hassan et al., 2018). Thus, this gap between women and men may be decreasing.

Results of the association between weight perception and age, SES, and educational level confirmed what was also already previously reported in non-Chilean populations (Freigang et al., 2020), indicating that men, low-income households, and groups with low educational status showed higher rates of misperception in adult samples (Alwan et al., 2010; Johnston and Lordan, 2014; Hassan et al., 2018; Pool et al., 2019). In all these cases, the trend is to underestimate their weight. The same phenomenon occurs with age. In this case, as people get older, weight underestimation increases (Park et al., 2019).

This patterning could suggest some socio-sanitary influences. Education status is related to health literacy (Garcia-Codina et al., 2019; Nutbeam and Lloyd, 2021). Consequently, educated people are expected to be more accurate about their weight status. The same occurs for higher income groups who might have more access to the healthcare system and, therefore, have their nutritional status monitored or warned about the risk of excess weight (Song et al., 2014). Another proposal is presented by Johnston and Lordan (2014) who suggested that since people often rely on comparison with peers to make assessments of their weight status, obesity has become the norm within low-income groups, and, unfortunately, it is undetected.

### Weight perception and weight management practices

Practices to lose weight, either following a diet or exercising, were generally not frequent in the sample, with dieting being more frequent than exercising. In a population-based study in Canada, more than half of the respondents reported a weight-loss attempt in the past 12 months (Raffoul and Hammond, 2018). In this study, and as expected, the percentage engaging in weight control activities increased as BMI increased and as perceived overweight/obesity also increased.

Actions to lose weight, dieting, or exercising, in participants with overweight and obesity were higher in those who had the right perception of their overweight/obesity condition. In contrast, the proportion of normal-weight people who declared attempts to lose weight was higher for those who perceived themselves as overweight than those with the correct perception of their weight. These findings suggest that in all groups, regardless of their real weight, weight loss behaviors were more related to the perceived nutritional status than the BMI-based nutritional status. In their review, Haynes et al. (2018) found strong evidence of the association between perceived overweight and weight loss attempts from cross-sectional studies. The same was found in a sample of young American men that informed more likelihood of reporting weight loss attempts in men who perceived themselves as overweight (Pool et al., 2019). In another research, participants with weight misperception had 85% lower odds of attempting weight loss than those with accurate weight perception (Hassan et al., 2018), while a population-based cross-sectional study that included 16,720 people concluded that weight control was positively associated with overweight perception (Yaemsiri et al., 2011). Based on the above, we might conclude that weight perceptions act as a starting point for weight control management and initiate any strategy for weight management. As a result, an accurate body weight self-perception is needed.

### Does accurate body weight perception suppose risks?

Previous research has stated some risks related to the perception of weight excess, asserting that “knowing hurts” (Robinson et al., 2017) and perceiving oneself as overweight might turn into psychological problems. For example, there is evidence that individuals who perceived themselves as overweight or obese showed a higher likelihood of mental health problems, particularly depression, than people who perceived themselves as about the right weight or reported their BMI as normal (Darimont et al., 2020). Also, Christoph et al. (2018) found that adolescents who perceived themselves as overweight (across all weight-status categories), compared with those who did not, showed higher internal mental distress and lower mean levels of psychosocial protective factors (such as positive identity, friend connectedness, and social competency) (Christoph et al., 2018). Additionally, being overweight was associated with a higher likelihood of engaging in unhealthy weight-loss methods (Raffoul and Hammond, 2018) and disordered eating (Haynes et al., 2018) compared with those who misperceived their weight status.

As already stated, the perception of being overweight prompts efforts in the search for a normal weight condition, such as dieting or asking for help from health providers (Yaemsiri et al., 2011; Johnston and Lordan, 2014; Dey et al., 2019). However, at the same time, recent research has asserted that the perception of being overweight was not reliably associated with physical activity or healthy dieting (Duncan et al., 2011), and even more, it predicts future weight gain. In their review, Haynes et al. (2018) concluded that individuals who perceived themselves as overweight were more likely to gain weight over time than those who perceived themselves as normal weight. A longitudinal study in three population-based surveys concluded that participants who perceived their weight status as being overweight were at an increased risk of subsequent weight gain (Robinson et al., 2015). In the same way, a prospective study that analyzed the weight change between 1996 and 2008 in 2,783 youth with obesity concluded that weight misperception predicted lower future weight gain (Sonneville et al., 2016). International trends support the notion that although weight misperception is decreasing, obesity is increasing, as the case of Danish data, which revealed that between 1995 and 2008, the prevalence of obesity increased and, in contrast, the misperception of weight status decreased (Matthiessen et al., 2014). The latter suggests that the perception of obesity is not always related to effective weight loss.

Some authors suggest that the link between the perception of obesity and weight gain is related to weight stigma (Robinson et al., 2017). In two cross-sectional studies conducted by Romano et al. (2018), participants who perceived their weight as overweight reported greater weight stigma concerns than participants who perceived their weight as about right. In addition, weight stigma concerns explained more than half of the variance in the relationship between perceived overweight and overeating trends. Being perceived as part of a stigmatized group induce psychological distress (Alimoradi et al., 2020), anxiety, antisocial behavior, and substance use (Papadopoulos and Brennan, 2015); therefore, what the authors suggest is that self-identification as overweight person place people at a greater risk of stress-induced overeating (Robinson et al., 2017), such as binge eating behaviors or emotional eating (Wu and Berry, 2018), and less healthy eating behaviors (Vartanian and Porter, 2016). These results may help to avoid what Major et al. (2014) identified as “the ironic effects of weight stigma.” In their study, they found that social media news aimed at fighting obesity can have paradoxical and unwanted effects, as women exposed to weight-stigmatizing news caused those who perceived themselves as overweight to consume more calories and feel less capable to control their eating behavior than when exposed to non-stigmatizing news. In a similar vein, Rivera and Paredez (2014) found that individuals who were highly self-stereotyped (i.e., had a self-concept of stigmatized ethnic-racial individuals) had lower levels of self-esteem than those who self-stereotyped less, which in turn predicted higher levels of BMI. Since that self-esteem is a valuable psychological resource that helps prevent overweight and obesity, self-stereotyping oneself as a member of a stigmatized group (being overweight) may accentuate negative social identity and contribute to increased overeating and unhealthy, disordered eating. This finding should draw attention to the added effects of double stigmatization when overweight self-categorization is combined with membership in a minority or disadvantaged group.

Another proposal is that overweight perception is related to body dissatisfaction (Sonneville et al., 2016), which has been consistently related to binge eating and weight gain over time (Wardle et al., 2001; Stice et al., 2002; Lewer et al., 2016). This has been supported by the emotional eating proposal that poses excessive food intake as a form to cope with negative feelings (Evers et al., 2010; Wu and Berry, 2018). Emotional eating is conceived as a maladaptive behavior that operates as a momentary response to alleviate or regulate negative emotions (Tice et al., 2001) but usually leads to different health problems such as weight gain and eating disorders (van Strien et al., 2013; Frayn et al., 2018).

A proposition about the relationship between perception and weight gain, stated in this study, is that it is not the right weight perception that induces weight gain but rather the strategies people usually employ to lose weight. Perceiving oneself as being overweight is not harmful on its own since it is necessary to be aware of the weight condition to take action. However, those who self-perceive as overweight may employ maladaptive weight-loss strategies, such as unsustainable diets or restraining eating.

Evidence on restraining eating has shown that it is a significant risk factor for overeating and binge eating. Prior evidence suggests that when there is strict cognitive control over eating, this control is diminished, for example, due to a negative experience or stress, episodes of disinhibition and overeating are induced, in what is called the disinhibition effect (Van Strien, 2020). Emotional discomfort can act diminishing self-control strength, inducing disinhibited eating and elevated food intake (Ward and Mann, 2000).

### Strengths and limitations

This is the first study, as far as we know, to address this issue in a nationally representative sample of the Chilean population and also to provide some insight into the reasons behind the increasing trends of obesity in Chile. Although the BMI has some pitfalls, it is the most extended nutritional status measure and was classified differently between adults and older people in this study. As a cross-sectional study, it does not allow for an inferred causal relationship among the variables studied.

### Practical implications

Results pointed out some public health implications. Under-detected obesity might restrict intentions and behavior to control the risks associated with obesity, such as searching for treatment and nutritional advice. However, it is important to note that self-perceived as obese, a highly stigmatized group, might have important consequences. Although not explored in this study, this fact can be detrimental to mental health and induce negative practices such as damaging eating behavior and unhealthy weight control practices.

This places challenges for practitioners and the health systems to deliver programs to develop healthy and sustainable weight management programs.

## Conclusion

Half of the total sample perceived their nutritional status according to BMI-based nutritional status, with a higher tendency to underestimate their body weight, particularly in people with excess weight. In general, accuracy was low and higher in normal-weight people, women, groups from urban areas, and those with higher educational levels and higher SES than their counterparts. Compared with participants with a right perception of their nutritional status, those who overestimated their nutritional status showed a higher likelihood of following a diet or exercising to lose weight.

The effects of weight perception on psychological experience and body weight are discussed and need to be further explored.

## Data availability statement

Publicly available datasets were analyzed in this study. This data can be found here: http://epi.minsal.cl/bases-de-datos/.

## Ethics statement

The studies involving human participants were reviewed and approved by the Pontificia Universidad Católica de Chile. The patients/participants provided their written informed consent to participate in this study.

## Author contributions

All authors listed have made a substantial, direct, and intellectual contribution to the work, and approved it for publication.
